# Phase and antigenic variation govern competition dynamics through positioning in bacterial colonies

**DOI:** 10.1038/s41598-017-12472-7

**Published:** 2017-09-22

**Authors:** Robert Zöllner, Enno R. Oldewurtel, Nadzeya Kouzel, Berenike Maier

**Affiliations:** Department of Physics, University of Cologne, Zülpicher Str. 77, 50539 Köln, Germany

## Abstract

Cellular positioning towards the surface of bacterial colonies and biofilms can enhance dispersal, provide a selective advantage due to increased nutrient and space availability, or shield interior cells from external stresses. Little is known about the molecular mechanisms that govern bacterial positioning. Using the type IV pilus (T4P) of *Neisseria gonorrhoeae*, we tested the hypothesis that the processes of phase and antigenic variation govern positioning and thus enhance bacterial fitness in expanding gonococcal colonies. By independently tuning growth rate and T4P-mediated interaction forces, we show that the loss of T4P and the subsequent segregation to the front confers a strong selective advantage. Sequencing of the major pilin gene of the spatially segregated sub-populations and an investigation of the spatio-temporal population dynamics was carried out. Our findings indicate that pilin phase and antigenic variation generate a standing variation of pilin sequences within the inoculation zone, while variants associated with a non-piliated phenotype segregate to the front of the growing colony. We conclude that tuning of attractive forces by phase and antigenic variation is a powerful mechanism for governing the dynamics of bacterial colonies.

## Introduction

The contributions of genetic heterogeneity to the pathology, host persistence, transmission, and spread of bacterial pathogens is an expanding area of research relevant to a diverse range of species^1–4^. In particular, the processes of phase variation and antigenic variation render specific genes hypermutable and thus generate micro-heterogeneity within bacterial populations^5^. In contrast to a loss of mismatch repair causing hypermutations within the entire genome^6^, these hypermutations are “localised” to a subset of genes. While bioinformatic analysis reveals putative phase-variable genes in many bacterial species, only few have been studied for their functions^3^. Some bacterial species show mutations at rates of up to 10^−3^ per generation in genes encoding for surface-exposed structures^7^. Variation in surface structures has the potential to change the strength of bacteria-bacteria interactions^8–10^ and as a consequence to influence the shape of biofilms. However, little is known about its role for adaption dynamics of biofilms.

**Figure 1:**
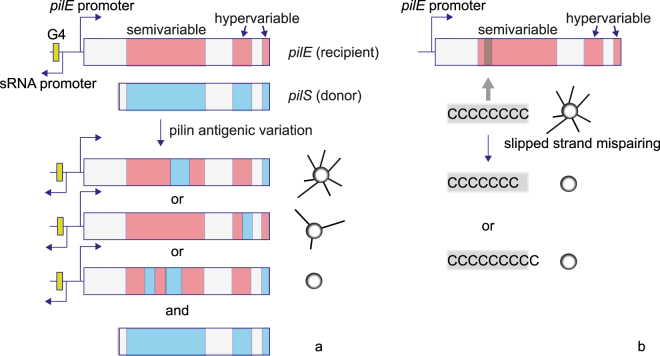
Pilin antigenic and phase variation. (**a**) Antigenic variation. The gene encoding for the major pilin subunit *pilE* contains semi- and hypervariable regions. Silent, promoter-less copies of the pilin, *pilS*, containing different sequences can partially recombine into the *pilE* recipient. This process depends on the G4 motif and *recA*. The pilin sequence of the resulting variant is modified and often non-functional, generating non-piliated variants^41^. (**b**) Phase variation due to homopolymeric repeats. Various genes involved in T4P biogenesis and post-translational modification contain homopolymeric stretches within their ORFs. Through slipped-strand mispairing during replication, the homopolymeric stretch is extended or truncated by a nucleotide at high probability and the gene is switched off.

In the case of the human pathogen *Neisseria gonorrhoeae* the major surface structures are extracellular polymers named type IV pili (T4P). This polymer consists of the major pilin subunit PilE and multiple minor pilins and mediates cell-cell interaction^11^. Modulation of T4P density or structure has been shown to affect the shape of early biofilms via a cell sorting mechanism^12^. Multiple genes related to the biogenesis or post-translational modification of T4P are phase-variable^5,13^. In particular, *pilE*, the gene encoding for the major pilin subunit, can contain a poly-C stretch where addition or excision of one base causes a frameshift mutation^10^ switching the gene off (Fig. 1). Furthermore the primary sequence of PilE undergoes high variation as a result of gene conversion (pilin antigenic variation) via partial recombination from silent pilins, *pilS* genes, into the *pilE* gene^5^ often leading to non-functional pilins and thus non-piliated bacteria (Fig. 1)^14^. This process of pilin antigenic variation is dependent on *recA* and a guanine quadruplex motif (G4) upstream of *pilE* ^15^. A high degree of variation in T4P could be observed during experimental human infections with gonorrhea with multiple *pilE* variants being present at each point of time. These showed dynamic sequence variations, that likely occurred via recombination with silent copies^16,17^. While antigenic variation is known to facilitate escape from immune surveillance, the roles of pilin antigenic variation and of phase variation in gonococcal biofilms are currently unclear. In several bacterial species, T4P determine the structure and dynamics of biofilms^18–22^. It is tempting to speculate that phase and antigenic variation in surface-related genes generate genotypic heterogeneity that enables bacteria to influence their position within biofilms.

Here, we address genetic mechanisms that generate subpopulations with differential cell-cell interaction forces. Moreover, we study how this impacts on bacterial fitness within an evolving colony, which serves as a model-biofilm. In a first set of experiments, we show that loss of piliation and subsequent segregation confers a strong selective advantage. Then, we investigate how pilin phase and antigenic variation affect the population dynamics of colony formation. Our results are most consistent with phase and antigenic variation setting up a standing variation of interaction forces within the inoculation zone. Then pilin variants with lowest interaction forces are selected for by being positioned to the front of the expanding colony.

## Results

### Slowly growing, weakly interacting cells surf on the expanding front of fast growing, strongly interacting cells

We have previously found that differential interaction forces between bacteria cause cellular sorting^12^. In particular, we modified the type IV pilus (T4P) of *N. gonorrhoeae* for tuning the bacterial interaction forces. The strongest phenotype was found when bacteria lost the ability to generate pili; the interaction between non-piliated (P−) cells was reduced to an undetectable level. As a consequence, the P− cells segregated to the front of expanding microcolonies^12^. In the following, we address the question how positioning affects the relative fitness of strongly interacting piliated and weakly interacting non-piliated cells within a growing colony acting as a model biofilm.

To this end, we designed bacterial strains that allowed us to independently tune the exponential growth rate and the bacterial interaction forces (Fig. S1a). Strain *red ermC*+ and *green* ^*Q*^ *ermC*− carry *mcherry* and *gfpmut3* genes, respectively, under control of the strong *pilE* promoter, and are neighboured by the *ermC* gene controlled by its own promoter encoding for the 23 S RNA methylase conferring resistance against erythromycin. They are designed to confer either increased resistance due to the joint activity of both promoters, denoted by *ermC*+, or to confer resistance only by the *ermC* promoter, denoted by *ermC*−. Additionally, the gene for the T4P secretin *pilQ* was deleted in *green* ^*Q*^ *ermC*− rendering it non-piliated. Previously, we have shown that deletion of *pilQ* increased the generation time only marginally^12^.

For non-interacting cells in unconstrained and non-limiting growth conditions the subpopulation with the lowest generation time, i.e. the subpopulation with the highest bacterial fitness, increases in frequency with time^23,24^. In the following, we will use the generation times of the strains obtained during exponential growth conditions and use their ratio to estimate the relative growth rates of the strains competing during colony growth. The generation times in the exponentially growing phase were determined on agar plates following a previously established protocol^12^.

**Figure 2:**
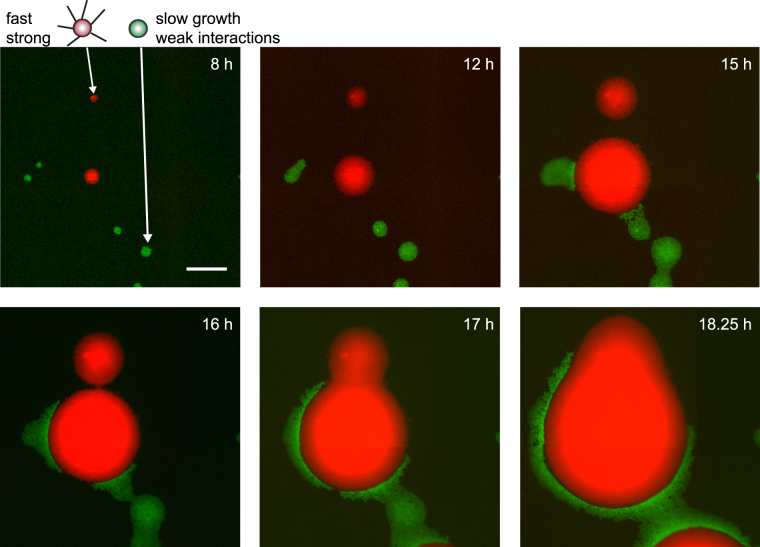
During exponential phase, slowly growing non-piliated bacteria surf on the front of a fast growing piliated colony. Time lapse of expanding colonies formed by slowly growing/weakly interacting *green* ^*Q*^ *ermC*− (Ng118, green) and fast growing/strongly interacting *red ermC*+ (Ng065, red) grown on 4 µg/ml erythromycin. *r*_*t*_ = *t*_*gen*_(*green* ^*Q*^ *ermC*−)/*t*_*gen*_(*red ermC*+) = 1.9. Brightness and contrast were adjusted differently for each image. Scale bar: 100 µm.

Using these strains, we assessed whether non-piliated cells were able to stay at the front of growing colonies despite their strongly increased generation time. Non-piliated *green* ^*Q*^ *ermC*− and piliated *red ermC*+ were inoculated onto an agar plate containing [erm] = 4 µg/ml; resulting in a ratio between their generation times of *r*_*t*_ = *1.9* (Fig. S1b). Under these conditions, exponential growth was observed for 10 hours. When fast growing, mostly piliated *red ermC*+ colonies impinged on slow-growing non-piliated *green* ^*Q*^ *ermC*−, the non-piliated cells were pushed forward by the faster growing piliated cells (Fig. 2). Gene surfing describes the process how mutants occasionally stay at the front of an expanding population and increase in frequency despite being neutral or even deleterious^25,26^. In a similar manner, we will use the term surfing to describe the displacement of cells by other cells such that the displaced cells stay at the front of the expanding colony. In other words, the cells lacking cellular interactions were able to surf on the front of the faster growing strongly connected cells.

In summary, weakly interacting cells surfed on the front of expanding strongly interacting cells despite a decrease in relative growth rate by a factor of two.

### Weakly interacting/slowly growing cells dominate the expanding front of colonies

Figure 2 demonstrates that weakly interacting cells can surf on the front of strongly interacting cells despite strongly reduced exponential growth rate. Next, we addressed how the combination of frontal positioning due to loss of interaction forces and varying relative exponential growth rates affect competition dynamics in the colony.

**Figure 3:**
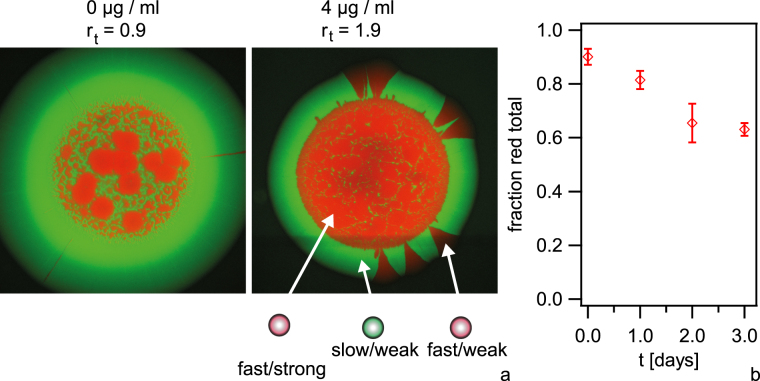
Competition between initially strongly interacting *red ermC*+ (Ng065) and weakly interacting *green* ^*Q*^ *ermC*− (Ng118) cells at varying exponential growth rates. (**a**) Examples of mixed colonies (inoculation ratio *r*_*in*_ = [*red ermC*+]/[*green* ^*Q*^ *ermC−*] = 10) after 68–70 h at varying concentrations of antibiotics. *r*_*t*_ = *t*_*gen*_(*green* ^*Q*^ *ermC*−)*/t*_*gen*_(*red ermC*+). Scale bar: 500 µm. (**b**) Fraction of *red ermC*+ within entire colony as a function of time obtained from single cell counts of red and green fluorescing cells. *r*_*in*_ = *10, r*_*t*_ = *1.9*. Error bars: Standard deviations obtained from three independent experiments, N > 100 cells for each time point and independent experiment.

To this end, we inoculated droplets containing piliated *red ermC*+ and non-piliated *green* ^*Q*^ *ermC*− at varying inoculation ratios of *r*_*in*_ = [*red ermC*+]: [*green* ^*Q*^ *ermC*−] = 10 onto agar plates containing different concentrations of erythromycin (Fig. 3a, Fig. S2). Within the initial inoculation zone the *red ermC*+ tend to form round patterns surrounded by *green* ^*Q*^ *ermC*− in agreement with Fig. 2. After (68–70) h of growth, *green* ^*Q*^ *ermC*− dominated the outgrowth (Fig. 3a, Fig. S2a). We investigated whether the total fraction of *red ermC*+ within the expanding colony decreased as a function of time since this decrease would indicate that *green* ^*Q*^ *ermC*− have a fitness advantage. Thus, we counted the fraction of *red ermC*+ within entire colonies inoculated at *r*_*in*_ = 10*, r*_*t*_ = 1.9 as a function of time by imaging individual cells on the mircoscope. The fraction of *red ermC*+ decreased with time, confirming that the non-piliated cells have a selective advantage despite having a lower exponential growth rate (Fig. 3b).

Interestingly, we sometimes found red sectors in the area of outgrowth (Fig. 3a). We suspected that these sectors were formed by the offspring of single mutants that had switched from the piliated to the non-piliated state. The sector density was independent of the ratio of generation times *r*_*t*_ (controlled by the concentration of erythromycin) but increased with the inoculation ratio from *r*_*in*_ = 1 to *r*_*in*_ = 100 (Figs S2 and S3). This is consistent with red sectors arising by loss of pili as a consequence of mutation. We used immunofluorescence against pili to verify that the red sectors were indeed non-piliated (Fig. S4). Furthermore, we quantified the area fractions of red cells *f*_*red*_ (non-piliated variants) in the outgrowth (Fig. S2). In the absence of erythromycin *f*_*red*_ remained constant in accordance to comparable exponential fitness of both strains, while with increasing concentrations of erythromycin, the area fraction *f*_*red*_ increased, in agreement with a higher relative growth rate. Notably, for colonies inoculated with *r*_*in*_ = 1 and *r*_*in*_ = 10 the non-piliated green cells still dominated the outgrowth after three days of competition despite a nearly 2-fold reduction in growth rate.

We conclude that benefits resulting out of changes in cell-cell interactions are sufficient to compensate considerably lower exponential growth rates. Non-piliated mutants of initially strongly interacting cells form sectors at the expanding front ensuring their survival.

### Naturally occurring non-piliated mutants sort to the expanding front and their offspring dominates the population

**Figure 4:**
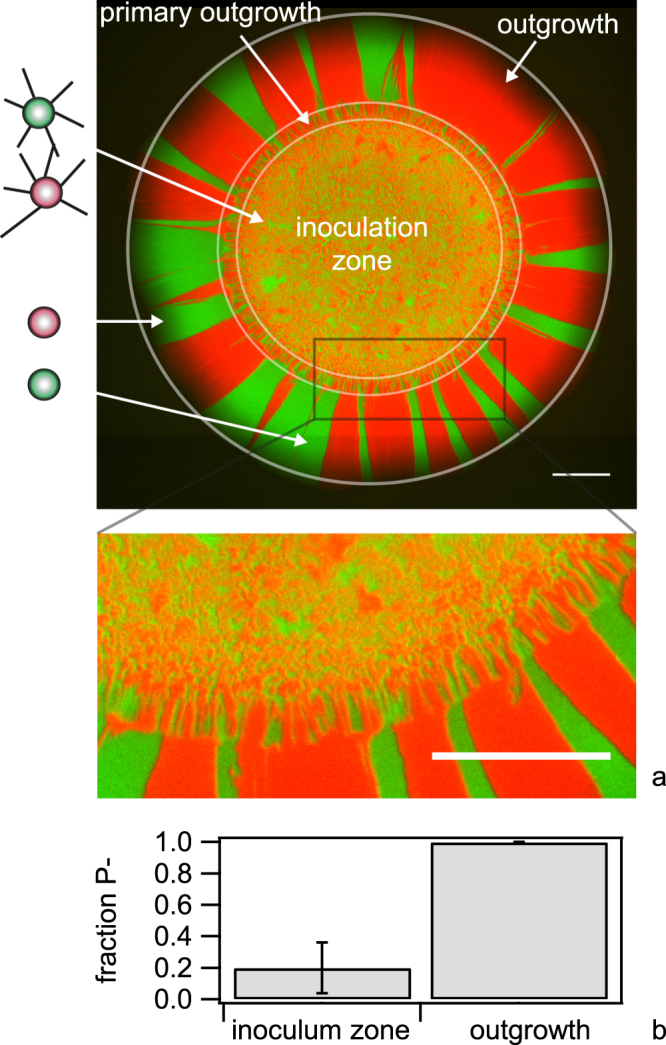
Pattern of wt colony. A droplet containing a mix of *green wt* (Ng165) and *red wt* (Ng106) bacteria was applied to an agar plate. (**a**) The colony was imaged using fluorescence microscopy (here after 48 h). Scale bar: 500 µm. (**b**) Cells were picked from the outgrowth and inoculation zone, respectively, diluted in medium and subsequently inoculated onto an agar plate. From the colony morphologies grown on the plate, the fraction of non-piliated cells was determined. N > 150 colonies for each condition. Error bars: standard error from three independent experiments.

We studied the effect of loss of piliation on the dynamics of expanding gonococcal colonies. To this end, piliated strains still carrying the wt *pilE* gene, but also different fluorescent reporters in non-essential loci (*green wt* and *red wt*) were mixed at a ratio 1: 1 and a droplet of this cell suspension was inoculated onto an agar plate. The addition of these reporters has no significant effect on exponential growth rates^12^. After (48–49) h the colony was imaged (Fig. 4). Bacteria grew within the inoculation zone. In the areas of outgrowth, they generated sectors (primary outgrowth). Formation of sectors in an expanding colony has been reported previously for *Escherichia coli* ^27^. For gonococci, however, the pattern at the front changed abruptly after ~150 µm outgrowth. The number of sectors was strongly reduced, indicating that a few newly arisen clones had established at the front of the growing colony.

**Figure 5:**
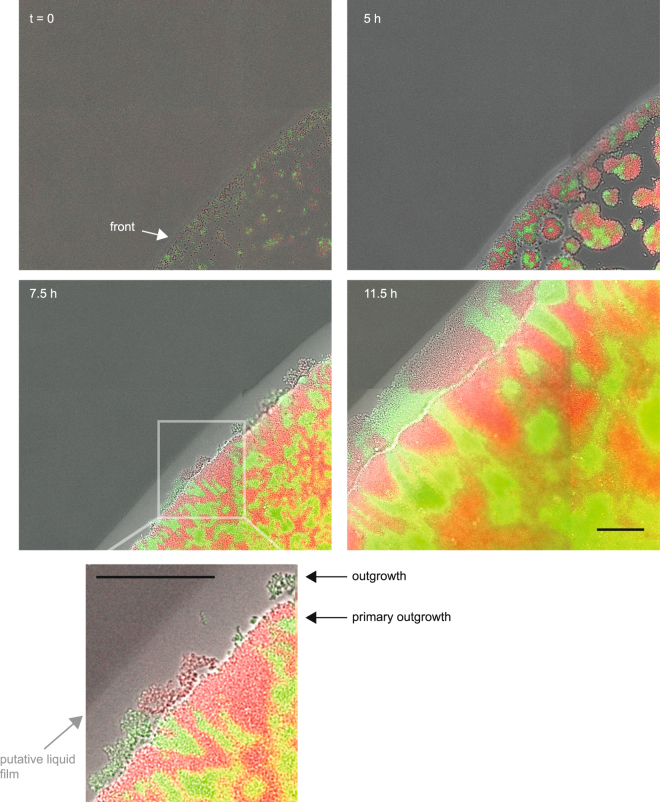
Dynamics of an expanding colony front. Time lapse of growing colony (region of interest of Movie S1). A droplet of piliated *red wt* (Ng106) and *green wt* (Ng165) was inoculated. Overlay between brightfield image and fluorescence images. Scale bar: 50 µm.

Using time lapse microscopy, we imaged the dynamics of colony expansion (Movie S1, Fig. S5). Growth starts from individual or small groups of bacteria (Fig. 5, Movie S2). Drying of the inoculum leads to an increased density of bacteria at the rim of the inoculated droplet compared with the remainder of the inoculation area^27^. The front closes after ~5 h of growth for this inoculation density. Early on, the outgrowth becomes discernible with a low density of bacteria appearing to be disconnected from the bulk of the colony (Fig. 5 inset). At this time, a light stripe becomes visible, growing with time. We think that the most likely explanation for this stripe is a liquid film formed on top of the agar plate^28^. The bacteria within the outgrowth form sectors whose pattern is hardly correlated with the pattern of the primary outgrowth. Expansion of the primary outgrowth is visible, but eventually slows down at ~15 h (Fig. S5).

The apparent low interaction of the cells within the outgrowth was reminiscent of a non-piliated (P−) phenotype. To check for piliation, we picked gonococci from the front of the expanding colony, diluted them in medium and inoculated them at low density onto agar plates such that each colony was most likely founded by a single cell. The resulting colonies showed the flat morphology characteristic of non-piliated (P−) gonococci^29^. Visual inspection of colony morphology has been routinely used for selecting between piliated P+ and non-piliated P− colonies^30^. Additionally, we verified our selection procedure using immunofluorescence with antibodies against the major pilin subunit PilE. While pili were clearly visible around bacteria selected for P+ colony morphology, the number of pili was severely reduced within the P− colonies indicating that the majority of bacteria in the outgrowth had lost their pili (Fig. S6). Subsequently, we quantified the fraction of colony forming units (CFU) showing a P− morphology. We found that ~20% of the CFU from the inoculation zone and nearly 100% of the CFU from the outgrowth had a non-piliated morphology (Fig. 4b).

**Figure 6:**
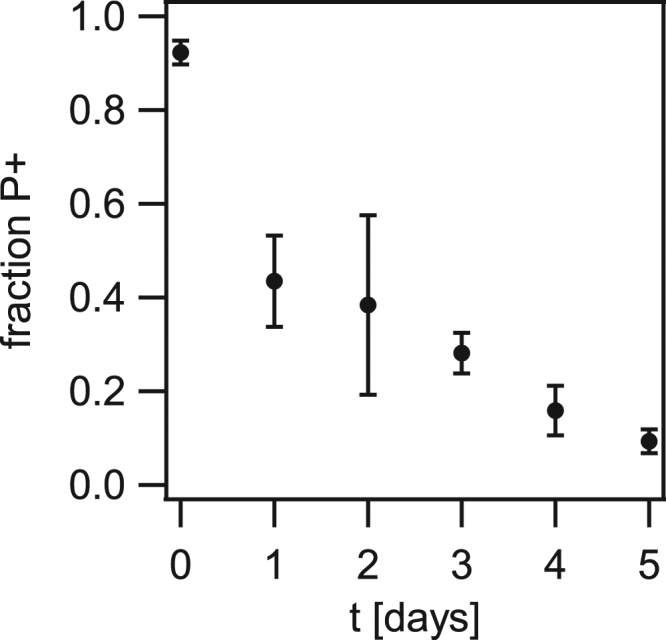
Loss of piliation confers selective advantage. (**a**) Droplets containing *green wt* (Ng165) bacteria were applied to an agar plate. Each day, a colony was suspended in liquid, diluted, and plated. The fraction between CFU with piliated (P+) morphology was determined. Error bars: standard deviation from three independent experiments.

To assess whether the non-piliated mutants had a selective advantage within the growing colony, we evaluated the fraction of P+ cells during colony growth. Droplets of wt P+ cells were inoculated as described before. At different time points, an entire colony was diluted in liquid medium and inoculated onto an agar plate for counting the number of piliated and non-piliated colonies, N_P+_ and N_P−_, via visual inspection of colony morphology. We found that within the initial inoculum at day 0, most cells were piliated with N_P+_/(N_P+_ + N_P−_) = 0.92 ± 0.03 (Fig. 6). Within one day of growth non-piliated cells dominated the population and the ratio increased continuously during several days of growth.

We conclude that gonococcal colonies contain a fraction of non-piliated cells and that non-piliated cells gain selective advantage by segregating to the expanding front.

### Pilin antigenic and phase variation govern the dynamics at the front of gonococcal colonies

The level of pilus production is highly variable due to phase variation and pilin antigenic variation in *N. gonorrhoeae* ^14^. In fact, bleb formation in expanding colonies has been developed into a tool for quantifying the probability of antigenic variation^31^. Here, we tested whether pilin antigenic variation and phase variation triggered cell sorting in expanding gonococcal colonies.

**Figure 7:**
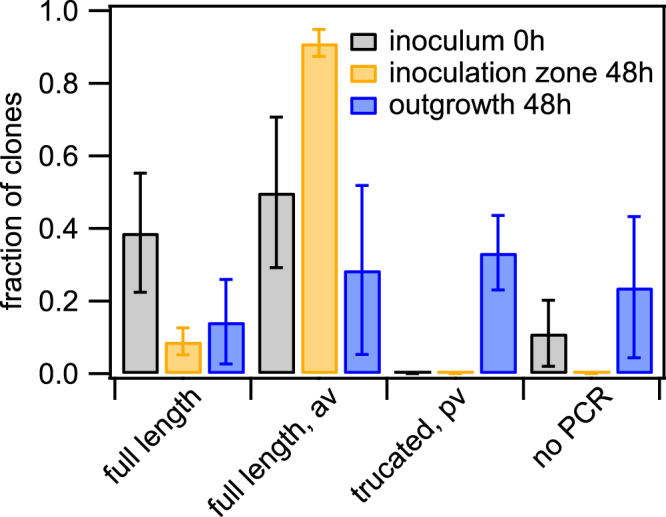
Pilin antigenic and phase variation. *red wt* (Ng106) and *green wt* (Ng165) were picked from the initial inoculum, the inoculation zone (48 h), and the outgrowth (48 h), respectively. After dilution and growth on agar plates, individual colonies (clones) were picked and *pilE* was sequenced. Sequences were categorised and the fraction shown is the number of sequences per category normalised by the total number of sequences of inoculum, inoculation zone and outgrowth, respectively. Full length: most abundant sequence in inoculum; full length, av: sequence change mappable to *pilS* sequence; truncated, pv: length change in poly-C sequence causing premature stop-codon; no PCR: PCR amplification did not result in a product. Mean and standard error of three independent experiments.

As a first step, the gene encoding for the major pilin subunit, *pilE*, was sequenced in multiple clones picked from the initial inoculum, the outgrowth, and the inoculation zone, respectively (see Methods). For comparison of the sequences, a multi-alignment tool was used. We categorised the mutations in *pilE* into four different categories and normalized by the total number of sequences from each picking site (Fig. 7). ‘Full length’ comprises all clones showing the most abundant sequence of the inoculum. ‘Full length, av’ includes all clones whose *pilE* sequences contain mutations mappable onto *pilS* sequences without having a premature stop codon. Therefore, these *pilE* sequence most likely encode for functional pilin. ‘Truncated, pv’: The ORF of *pilE* contains a homopolymeric stretch of eight cytosines within the semivariable region (Fig. 1b). Clones with seven or nine cytosines were pooled into this category. Since the resulting frameshift causes a premature stop codon, the mutations most likely result in a phase variation with *pilE* in the OFF state. Finally, a deletion of the *pilE* locus is likely, where the *pilE*-specific primer pair used for amplification and sequencing was unable to produce a PCR product (‘no PCR product’)^14^.

The wt inoculum was dominated by two *pilE* sequence variants, most likely due to inoculation of the two strains *green wt* and *red wt* (Fig. 7). The inoculation zone after 48 h was dominated by different full length *pilE* variants including sequences that could be mapped onto *pilS1c1*, *pilS1c4*, *pilS1c5*, *pilS5c1*, *pilS6c1*, and *pilS7c1*, respectively (Table S3). These sequences are in agreement with the finding that most CFU are piliated within the inoculation zone (Fig. 4c). Within the outgrowth, a variety of mutations were found in the *pilE* sequence. (14 ± 10) % of the clones showed parental sequence and (29 ± 23) % of the clones showed full length *pilE* sequences with mutations that could be mapped onto *pilS6c3*, and *pilS1c1*, respectively. The fraction of clones with full length pilin variants was significantly lower in the outgrowth compared to the inoculation zone (Fig. S7). Concomitantly, the fraction of clones showing a phase variation within the poly-C-stretch of *pilE* was significantly larger within the outgrowth compared to the homeland (Fig. S7). In particular, (33 ± 10) % of the clones showed phase variation within the poly-C-stretch of *pilE*, one clone contained an L-pilin sequence (tandem copy), and (24 ± 19) % of the clones did not yield a PCR product. Since 100% of bacteria are non-piliated within the outgrowth and the primer pair used for amplification and sequencing is specific to the *pilE* locus, an inability to produce a PCR product most likely indicates that the *pilE* ORF was lost^14^. Thus the majority of gonococci within the outgrowth contain either truncated pilin or loss of the pilin gene in agreement with the non-piliated phenotype. The presence of clones with the parental *pilE* open reading frame within the outgrowth indicates that processes other than pilin antigenic variation that affect *pilE* expression generate non-piliated bacteria. This is consistent with reports showing that phase-variation of T4P related genes causes loss of pili^14^.

**Figure 8:**
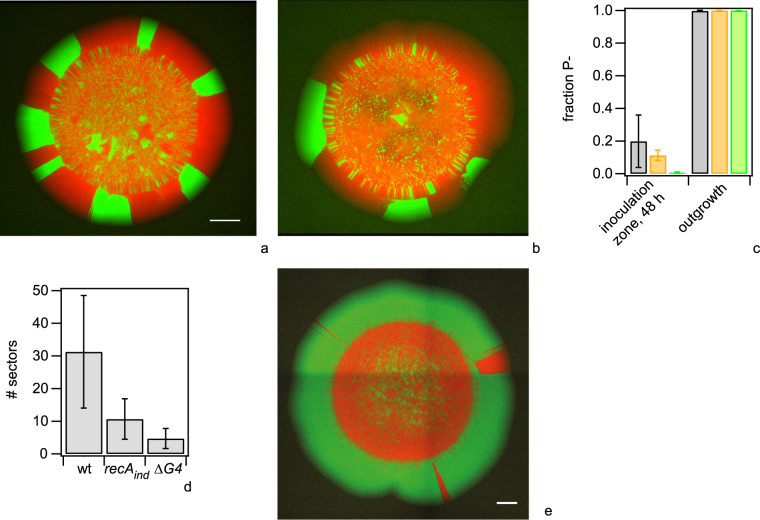
Pilin antigenic variation governs colony dynamics. (**a**–**d**) A droplet of differentially labeled strains was inoculated onto an agar plate and colonies were grown for 48 h. Typical colonies of (**a**) pilin antigenic variation deficient *green recA* (Ng167) and *red recA* (Ng168), and (**b**) pilin antigenic variation deficient *green ∆G4* (Ng169) and *red ΔG4* (Ng170). (**c**) Fractions of non-piliated cells in 48 h colony determined by colony morphology. Grey: wt, orange: *recA*_*ind*_, green: *ΔG4*. N > 150 colonies for each condition. Mean and standard error from three independent experiments. (**d**) Number of sectors in the area of outgrowth (N > 30 colonies for each condition. Error bars represent standard deviation). Distributions of numbers are significantly different for all pairs with P < 10^−4^ (t-test). (**e**) Effect of pilin antigenic variation on competition. A droplet containing a mix of *green wt* (Ng165) and antigenic variation deficient *red ΔG4* (Ng170) bacteria was applied to an agar plate. The colony was imaged using fluorescence microscopy after 48 h. Scale bars: 500 µm.

Next, we investigated whether cells that are incapable of pilin antigenic variation show different levels of piliation, colony morphology, colony dynamics, and distributions of pilin sequences. For this, we used *recA* inducible strains (without induction) and strains in which the G4 motif upstream of the *pilE* gene was deleted. Both *recA* and the G4 motif are essential for pilin antigenic variation^15,31^. We repeated the experiment described in Fig. 4; *green recA* and *red recA* were mixed at a 1:1 ratio and a droplet was inoculated onto an agar plate. The colonies were imaged after 48 h (Fig. 8a). The same experiment was performed with *green G4* and *red G4* (Fig. 8b). We found that the fraction of non-pilated cells (determined by colony morphology of CFU) from the inoculation zone after 48 h was ~11% for *recA*_*ind*_ and 0.6% for *ΔG4* (Fig. 8c). The *ΔG4* outgrowth shows a significantly lower fraction of non-piliated cells compared to both wt and *recA*_*ind*_ outgrowths (Fig. S8). While the average fraction of non-piliated cells was lower for the *recA*_*ind*_ strain compared to wt, the variance was too high to clearly support this difference. No piliated bacteria were detected in the outgrowth (Fig. 8c).

The colony morphology of the strains incapable of pilin antigenic variation was qualitatively similar compared to the wt, however the area of outgrowth (Fig. 8a,b) and the number of sectors within the outgrowth (Fig. 8d) were considerably lower. These observations are likely explained as follows. The fraction of non-piliated cells within the area of inoculation is lower and therefore we can expect that the onset of outgrowth formation is delayed compared to the wt colonies. Indeed, time lapse microscopy using *ΔG4* bacteria confirmed that the bacteria forming the outgrowth were detectable at considerably later time points compared to wt growth (Fig. S9). If the density of non-piliated cells at the front is lower, then fewer cells will form sectors and thus the sector density is lower in agreement with our findings (Fig. 8d).

Considering that the formation of the non-piliated outgrowth was delayed due to inhibition of pilin antigenic variation, we expect that the wt dominates the outgrowth when inoculated together with the antigentic variation inhibited *ΔG4* strain. To assess this expectation, we inoculated colonies from a mix of *green wt* and *red ΔG4*. Imaging the colonies after 48 h, showed that *green wt* dominated the outgrowth of colonies as expected (Fig. 8e). Since the exponential generation times of wt (49 min ± 3 min) and *ΔG4* (47 min ± 4 min) strains are not significantly different, we attribute the dominance of *green wt* within the outgrowth to more frequent loss of pili due to pilin antigenic variation.

**Figure 9:**
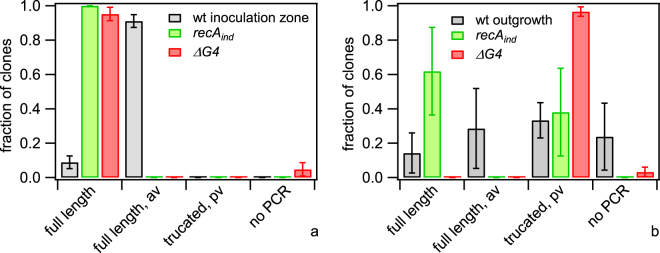
Pilin variation in antigenic variation deficient strains. Cells were picked from (**a**) inoculation zone, and (**b**) outgrowth, respectively. After dilution and growth on agar plates, individual colonies were picked and *pilE* was sequenced. Sequences were categorised and the fraction shown is the number of sequences per category normalised by the total number of sequences found for each strain. Full length: most abundant sequence in inoculum; full length, av: sequence change mappable to *pilS* sequence; truncated, pv: length change in poly-C sequence causing premature stop-codon; no PCR: PCR amplification did not result in a product. Grey: *red wt* (Ng106) and *green wt* (Ng165), green: *green recA* (Ng167) and *red recA* (Ng168), red: *green ∆G4* (Ng169) and *red ΔG4* (Ng170). Mean and standard error of three independent experiments.

To check whether pilin variation was involved in generating non-piliated gonococci using the *recA*_*ind*_ and *ΔG4* strains, we sequenced individual clones picked from the inoculum, the inoculation zone, and the outgrowth of colonies like those shown in Fig. 8a,b. For *recA*_*ind*_ and *ΔG4*, no variation was found in the inocula or the inoculation zone (Fig. 9a, Table S3, Fig. S10a). Both the *recA*_*ind*_ and the *ΔG4* outgrowth showed interesting differences compared to the wt outgrowth (Fig. S10). In particular, the outgrowth of the *recA*_*ind*_ strain was dominated by clones carrying the parental *pilE* sequence. (38 ± 26) % of the clones in the *recA*_*ind*_ outgrowth showed pilin phase variation (Fig. 9b). The fraction of the pilin phase variants within the *ΔG4* outgrowth of (97 ± 3) % was significantly higher than for the wt or *recA*_*ind*_ outgrowth (Fig. S10b).

In summary, mutations in the major pilin subunits via antigenic and phase variation lead to loss of pili and the accumulation of non-piliated variants in the outgrowth of expanding colonies. Inhibition of pilin antigenic variation affects the dynamics of colony spreading and the composition of *pilE* variants within the growing front.

## Discussion

### Role of pilin phase and antigenic variation in shaping biofilms

Phase and antigenic variation can serve several roles in bacteria, e.g. they have implications on setting up heterogeneity in clonal bacterial populations, escape from immune surveillance, nutrient acquisitions, motility, or gene regulation^1,2,4,32^. Here, we focus on the function of positioning in bacterial colonies. Positioning within structured environments crucially affects the competition dynamics between different subpopulations. In this work, we showed that pilin phase variation and pilin antigenic variation are important for the population dynamics in gonococcal colonies. Loss of piliation triggers sorting of non-piliated cells to the front of the expanding colony where they have a strong competitive advantage.

Pilin antigenic variation enhanced the density of non-piliated gonococci within the inoculation zone. As a consequence, the non-piliated outgrowth was generated by a larger number of non-piliated ‘founder’ cells and the piliated bacteria were enclosed more rapidly compared to expanding colonies incapable of pilin antigenic variation. Non-piliated cells still occurred in the absence of pilin antigenic variation, and were sorted to the expanding front. Interestingly, the two strains deficient for antigenic variation used in this study showed different compositions of pilin variants within the outgrowth. The outgrowth of the *ΔG4* strain showed almost exclusively pilin sequences with slipped-strand mispairing in the poly-C stretch. For the *recA*_*ind*_ strain, a subpopulation showed contraction or extension of the poly-C stretch, but most of the pilin sequences were not varied. Moreover, the density of non-piliated cells within the inoculation zone and the number of sectors within the outgrowth were higher for the *recA*_*ind*_ strain compared to the *ΔG4* strain. The fact that no recombination of *pilS* sequences or loss of the pilin ORF was found suggest, that the difference is not caused by leakiness of the *lac* promoter in the *recA* inducible strain. This observation is consistent with the assumption that mutations in other genes involved in pilus biogenesis generate non-piliated gonococci. Since *recA* is involved in DNA repair, its absence may result in a higher mutation rate. The most likely cause of pilin loss is phase variation of *pilC*, a gene required for T4P biogenesis^14,33^.

We conclude that pilin antigenic variation and phase variation generate a standing variation within gonococcal populations that govern bacterial positioning and competition dynamics.

### Different molecular mechanisms manipulate positioning within biofilms

Positioning has important effects on bacterial fitness in structured environments^26^. In the absence of external stresses, surface residing bacteria would benefit the most from unlimited space, high oxygen and nutrient concentration. In other cases positioning could help bacteria further inside the biofilm to evade stresses such as shear flow, antibiotics or immune cells. It is also plausible that relocating bacteria to the surface can act as a dispersal mechanism to spread cells to new biofilm attachment sites. Alternatively, dynamic positioning of cells within a biofilm could lead to cooperation of cells and help to avoid a static growth-arresting biofilm structure. It is therefore conceivable that bacteria have evolved mechanisms for manipulating physical interactions and thus for tuning their positions. For example, differential adhesion forces onto surfaces can affect fitness^34^. One example of a reversible mutational target associated with positioning is the *gac* regulon in *Pseudomonas fluorescens* ^35^. The regulon controls secretion and recent studies suggest that secretion of polymers pushes bacteria to the surface of biofilms^35^. At the expanding front of colonies, these mutants cooperate with a second mutant with altered levels of the second messenger cyclic di-GMP^36^. The combined physical properties of these two strains allow them to spread faster compared to the individual mutants; one strain provides a wetting polymer at the colony edge and the other strain pushes both along^36^. In the present work, we found that loss of type 4 pili confers a benefit because non-piliated bacteria segregate to the front of growing colonies. At the molecular level, loss of piliation strongly reduces the attractive force between bacteria generated by pili. Interestingly, both *P. fluorescens* and *N. gonorrhoeae* modulated local physical interactions within the biofilm-model of expanding colonies through mutations in specific genes. Therefore, it is tempting to speculate that altering the local physical interactions within biofilms through reversible mutations is a common strategy in biofilm adaptation.

### The role of differential physical interactions in genetic drift of structured bacterial populations

Computer simulations predict that bacterial clone surfing can occur in spatially structured, growing populations. In simulated expanding colonies, neutral and beneficial mutations arising near the edge could surf at the front leading to a larger abundance than would be expected in homogeneously mixed populations^37,38^. Furthermore, simulations show that even the offspring of deleterious mutations are expected to be present at higher frequency if they surf at the front^39^. Similarly, we show experimentally how strongly deleterious mutations occurring near the front, are displaced and show a surfing phenotype on the front of expanding colonies provided that they have weaker physical interactions than the competing variant. This raises the question whether variations affecting the bacterial interactions cause accumulation of deleterious mutations in other loci at the front of the expanding colony. In our experiments, the expanding front was not dominated by a single variant. Instead we found that the wt gonococci produce multiple variants that gain access to the front. They can be identified from differentially colored sectors in the area of outgrowth. Fig. S5, for example, shows sectors whose opening angle increases or decreases, respectively. This suggests that different variants with different fitnesses are present and compete with each other in the area of outgrowth. In a different environment, e.g. under flow conditions, we expect that non-piliated and weakly interacting piliated bacteria, would be dispersed and flushed away. Thus positioning cells towards the surface of the biofilm could provide an efficient mechanism to disperse cells to new biofilm attachment sites. For the cells that stay attached to the biofilm, differential interaction forces between T4P are likely to govern positioning. It is tempting to speculate that most events of pilin antigenic variation that produce functional T4P but with altered pilin sequences result in different cell-cell interaction forces. Multiple T4P-related genes are phase-variable in *N. gonorrhoeae*. They include genes encoding for enzymes that are involved in post-translational modification of pili^5,13^. The post-translational modification with disaccharides or phosphoform-modifications affects the interaction forces between gonococci and triggers sorting with respect to differential interaction forces^12^. Therefore, if the rate of phase and antigenic variation is sufficiently high, diversity increases and competition between variants is likely to antagonize the accumulation of deleterious mutations within the expanding front.

## Conclusion

The mechanisms of phase variation and antigenic variation of surface-related genes are known to help pathogens to survive when facing immune surveillance. Our experiments strongly suggest that pilin phase and antigenic variation have an additional function in generating standing variation of surface structures mediating differential bacterial interactions. In particular, we showed that the competition dynamics in gonococcal colonies is governed by the interplay between exponential growth rate, differential interaction forces, and pilin phase and antigenic variation. We took the approach of a colony growing on agar as a simple model biofilm to disentangle and focus on spatial interactions and growth rates. In a more complex biofilm system, a multitude of additional mechanisms is likely to influence bacterial fitness. Phase variation is also important for adaptation in other species including *Mycoplasmas*, *Campylobacters*, *Helicobacters*, *Bacteroides, Streptococcus*, and coliforms^1,40^. We propose that altering the local physical interactions within biofilms through phase and antigenic variation is an important and possibly common contributor to the dynamics of biofilm growth, dispersion and adaptation.

## Methods

### Bacterial growth conditions

*N. gonorrrhoeae* (Table S1) was grown overnight at 37 °C and 5% CO_2_ on agar plates containing gonococcal 1% base agar (wt/vol) and supplemented with 1% IsoVitaleX (vol/vol; BD Biosciences). Before each experiment gonococcal colonies were resuspended in GC-medium supplemented with 1% IsoVitaleX (vol/vol).

### Colony growth

Bacteria from overnight plates of each strain were re-suspended in GC medium to an optical density at 600 nm OD_600_ = 2.0. Suspensions of strains labelled either with a red or green fluorescent protein were mixed and droplets of with a volume of 0.25 µl were applied to agar plates containing 1% IsoVitaleX (vol/vol). Subsequently, the plates where incubated at 37 °C and 5% CO_2_. For competitions between weakly and strongly interacting cells, the suspensions were mixed in the ratios *r*_*in*_ = [*red ermC* + *recA*_*ind*_]: [*green* ^*Q*^ *ermC*− *recA*_*ind*_] = 1:1, 1:10, and 1:100, respectively, and the agar contained erythromycin concentrations of 0, 2, 3, and 4 µg/ml, as indicated in the figure captions. All other colonies were inoculated from suspensions of 1:1 ratios.

### Microscopy and image analysis

Agar plates carrying bacterial colonies were mounted onto an inverted epi-fluorescence microscope (Nikon Ti-E, Japan) equipped with a motorized stage and a custom-built thermo-box. The plate was fixed within a custom-built chamber with a glass bottom to allow for imaging and CO_2_ supply, keeping the plate under an atmosphere of 37 °C and 5.9% CO_2_.

Static images of colonies where taken with a 2×/0.06 air objective (Nikon). Time lapse imaging of growing colonies was done with a 40×/0.6 long-working distance air objective (Nikon) capturing images every 30 min over a grid of 208 different positions covering an area of 3.25 mm × 3.04 mm.

Time lapse imaging of growing microcolonies to determine exponential growth rates was done employing the 40×/0.6 objective (Nikon) capturing images every 15 min over a grid of 120 different positions covering an area of 2.3 mm × 2.5 mm.

In order to calculate area fractions in the area of outgrowth of colonies, the area of outgrowth was mapped to a linear image using polar transformation with ImageJ. Intensity profiles of the transformed images were measured over the full extent of the area of outgrowth at different radii using MATLAB. Threshold setting allowed for calculating area fractions of each profile.

The fraction of *red ermC*+ cells from entire colonies was determined by suspending an entire colony after (0–3) days of growth into GC medium. The suspensions were vortexed and subsequently, the number of cells showing a signal in the red and green channels of the fluorescence microscope, respectively, was counted. Three independent experiments were performed each with >100 cells imaged for each time point.

### Sequencing of *pilE*

In total 15–20 colonies of the inoculated population grown on agar plates overnight were picked for sequencing for each condition. Inoculated bacteria were grown on agar plates forming colonies with inoculation zone and outgrowth. After 48 h bacteria were picked from the outgrowth and inoculation zone and subsequently inoculated onto an agar plate. For each strain, greater than 20 individual colonies were picked for sequencing from the inoculation zone and outgrowth in each of three independent experiments. For sequencing of *pilE*, PILRBS (Sequence at ribosome-binding site of *pilE*) and SP3A (Sequence in 3′ conserved untranslated region of all pil loci) primers were used following Seifert *et al*.^17^ and Wright *et al*.^6^. The *pilE* fragment amplified by colony PCR was sequenced by a DNA sequencing service (GATC Biotech AG., Konstanz, Germany).

## Electronic supplementary material

Movie S1

Movie S2

Supplementary Information
