# Supplementary material for: Phase and antigenic variation govern competition dynamics through positioning in bacterial colonies
Source: Sci Rep. 2017 Sep 22;7:12151. doi: 10.1038/s41598-017-12472-7 (PMC5610331; doi:10.1038/s41598-017-12472-7)
Supplement: Supplementary file 3 — Supplementary Information [file 41598_2017_12472_MOESM3_ESM.pdf]

Supplementary Information for

Phase and antigenic variation govern competition dynamics through positioning in bacterial colonies

Robert Zöllner*[1], Enno R. Oldewurtel*[1], Nadzeya Kouzel[1], Berenike Maier[1§]

[1] Department of Physics, University of Cologne, Zülpicher Str. 77, 50539 Köln, Germany

Supplementary Materials and Methods

Detailed bacterial growth conditions. Gonococcal base agar was made from 10 g/l BactoTM agar (BD Biosciences, Bedford, MA, USA), 5 g/l NaCl (Roth, Darmstadt, Germany), 4 g/l K2HPO4 (Roth), 1 g/l KH2PO4 (Roth), 15 g/l BactoTM Proteose Peptone No. 3 (BD), 0.5 g/l soluble starch (Sigma-Aldrich, St. Louis, MO, USA)) and supplemented with 1% IsoVitaleX: 1 g/l D-Glucose (Roth), 0.1 g/l L-glutamine (Roth), 0.289 g/l L-cysteine-HCL×H20 (Roth), 1 mg/l thiamine pyrophosphate (Sigma-Aldrich), 0.2 mg/l Fe(NO3)3 (Sigma-Aldrich), 0.03 mg/l thiamine HCl (Roth), 0.13 mg/l 4-aminobenzoic acid (Sigma-Aldrich), 2.5 mg/l β-nicotinamide adenine dinucleotide (Roth) and 0.1 mg/l vitamin B12 (Sigma-Aldrich). GC medium is identical to the base agar composition, but lacks agar and starch.

Strain constructions. For tuning growth rates, gene constructs were created coding for a fluorescent reporter gene transcribed from the strong *pilE* promoter, neighboured in sequence by the *ermC* gene with its own promoter encoding for the 23S RNA methylase conferring resistance against erythromycin. Different constructs were made with the orientation of *ermC* either in the same direction as the reporter gene to achieve a higher activity, which we denote by *ermC*+, or with reversed orientation such that *ermC* denoted by *ermC*-. The *green ermC+* strain was constructed to contain both *gfpmut3* gene and *ermC* gene under the strong *pilE* promotor. The promoter region of *pilE* (P_{pilE}) with the upstream region of *igA1* locus was amplified from gDNA of gonococcal strain MS11 using primers NK202 and NK135 (Table S2). The *gfpmut3* gene was amplified from the *pIga::*P_{pilE}*gfpmut3* vector [1] using primers NK134 and NK204. The *ermC* gene and downstream region of *igA1* locus were amplified from the *pIga* vector [2] using primers NK203 and NK199. A PCR fusion between P_{pilE}, *gfpmut3* and *ermC* gene was generated using NK202 and NK199, yielding the final fragment P_{pilE}*gfpmut3 ermC*, where the *ermC* gene is expressed from its own promotor and additionally from a strong P_{pilE} promotor. The final DNA fragment was transformed in VD300, generating strain Ng117 *pIga::*P_{pilE}*gfpmut3 ermC*.

To enhance the fluorescent signal, we used the yellow fluorescent protein EYFP instead of GFPMUT3 in the strains used for the competition between piliated strains. The strain *iga::P$_{pilE}$eyfp ermC* with a yellow fluorescent reporter in the *igA1* locus was constructed as follows: the promoter region of *pilE* (P$_{\text{pilE}}$) with a SacI restriction site was amplified from gDNA of gonococcal strain MS11 using primers NK83 and NK8. *eyfp* with a SacI restriction site was amplified from the pEYFP-C1 vector (Clontech) using primers NK9 and NK48. A PCR fusion between P$_{\text{pilE}}$ and *eyfp* was generated using NK83 and NK48. The product was inserted into SacI site of p2/16/1 [2], resulting in the pIga::P$_{\text{pilE}}$eyfp plasmid. This plasmid was used to introduce the eyfp ermC alleles into the *iga* locus of strain VD300, generating strain Ng165 *iga::P$_{pilE}$eyfp ermC*.

The *green* Δ*G4* and *red* Δ*G4* strains were constructed as follows: primers NK78 and NK180 were used to amplify the 937 bp DNA region upstream of guanine quartet (G4) motif and primers NK179 and JB109 were used to amplify the 440 bp DNA region downstream of G4 motif, including the *pilE* promotor and part of *pilE* gene. Primers NK181 and NK182 were used to amplify *aac*, encoding for apramycin acetyl transferase together with its promotor region from the pUP6ΔcomE1 vector (Gangel et al., 2014).

The upstream and downstream DNA regions of the G4 motif were fused to the 5' and 3' apramycin resistance gene, respectively. The resulting DNA fragment was spot transformed in VD300, creating strain Ng150 Δ*G4*. For this, gonococcal cells were co-incubated with DNA in one spot on a GC-plate. The resulting colonies were re-suspended in GC-medium, spread onto selective antibiotic plates and incubated overnight. Subsequently single colonies of transformants could be picked. The Δ*G4* mutation was verified by PCR and sequencing analysis. *eyfp* and *mcherry* genes were introduced to this mutant using *pIga::P$_{pilE}$eyfp and pLAS::P$_{pilE}$ mcherry* plasmids respectively, leading to the Ng169 *green* Δ*G4* and Ng170 *red* Δ*G4*.

Determination of generation times. We used a previously published protocol for determining generation times of gonococci [3]. Optical density measurements failed in our hand for characterization of growth rates, most likely because gonococci tend to form clusters and attach to the surface even under vigorous shaking. Single colonies from overnight plates of each strain were re-suspended in 2 ml GC medium. A droplet of 5 µl of the mixed suspension was used to inoculate agar plates containing 1% IsoVitaleX and erythromycin at concentrations of 0, 2, 3 and

4 µg/ml, as indicated in the figure captions. The low density of cells in the inoculum ensured that exponential growth of single colonies could be monitored. The rate of fluorescence increase of the growing colonies was used to measure the generation time. A detailed description is given in a previously published protocol [3].

We note that the absolute generation times varied slightly between different days. Therefore, for comparing generation times of different strains, they were always grown on the same agar plates and subsequently data from at least three different days were averaged.

Growth rates. We found that the generation times of *red ermC+* and *green^Q ermC-* were comparable in the absence of antibiotics (Fig. S1b). With increasing concentration of erythromycin, the generation time of strain *red ermC+* increased only slightly while the generation time of strain *green^Q ermC-* increased from t_{gen} = (58 ± 1) min at [erm] = 0 µg/ml to t_{gen} = (130 ± 1) min at [erm] = 4 µg/ml (generation times given as mean ± standard error).

To verify that the increased transcription of the *ermC* gene by the activity of the *pilE* promotor was responsible for the different growth rates in the presence of erythromycin, we reversed the direction of *gfpmut3* in the *gfpmut3*-expressing strain (*green ermC+*) (Fig. S1a). Now the generation times of *green ermC+* and *red ermC+* at [erm] = 4 µg/ml were comparable (Fig. S1b), confirming that the orientation of *ermC* with respect to the *pilE* promotor affects the generation time in the presence of erythromycin.

Immunofluorescence. Bacteria from GC-plates were diluted in PBS and fixed on coverslips with 4% Para-Formaldehyde (PFA). The samples were blocked with PBS (0.5% BSA) and subsequently incubated with antibody against PilE (1 : 100) in PBS (0.5% BSA). They were then incubated TexasRed-conjugated secondary antibody (1 : 500) in PBS (0.5% BSA) while being kept covered in the dark. In between the incubation steps the coverslips were washed carefully with PBS (0.5% BSA) and MillQ. The samples were imaged with with a 100 x oil immersion objective (Nikon) on an inverted microscope (Ti-E, Nikon).

Fisher's exact test. For figures 7, 8c, and 9, we used Fisher's exact to assess the significance of differences between different conditions or strains. We generated contingency tables where the rows contained data from different strains (Figs. 8c, 9) or locations / time points (Fig. 7) and the columns contained the number of clones within the category of interest and the number of clones within all other categories. We used the Fisher's exact test implemented in MatLab on these 2 x 2 matrices to calculate the significance level p indicating the probability, that the difference between the values depends on the different strains or conditions. The pair-wise p-values are plotted in Figs. S7, S8, and S10.

Supplementary Tables

Strain	Relevant genotype	Source/Reference
VD300 (Ng002)	wild-type, *opa*- selected	[4]
N400 (Ng003)	*recA6ind*(*tetM*)	[5]
red ermC+ (Ng065)	*igA1*::*PpilE mcherry ermC* *recA6ind*(*tetM*)	This study
green ermC+ (Ng117)	*igA1*::*PpilE gfpmut3 ermC* *recA6ind*(*tetM*)	This study, [1]
green^{Q} *ermC-* (Ng118)	*pilQ*::*m-Tn3cm* *igA1*::*PpilE gfpmut3 ermC* *recA6ind*(*tetM*)	[3]
green wt (Ng165)	*igA1::PpilE eyfp ermC*	This study
red wt (Ng106)	*lctP*:*PpilE mcherry aadA*:*aspC*	This study
green recA (Ng167)	*igA1::PpilE eyfp ermC* *recA6ind*(*tetM*)	This study
red recA (Ng168)	*lctP*:*PpilE mcherry aadA*:*aspC* *recA6ind*(*tetM*)	This study
green Δ*G4* (Ng169)	*igA1:: PpilE eyfp ermC* Δ*G4*	This study
red Δ*G4* (Ng170)	*lctP*:*PpilE mcherry aadA*:*aspC* Δ*G4*	This study

Table S1 List of strains

**Primers**	**Sequence 5’ – 3’**
NK8	TCAATTAGGAGTAATTTTATGGTGAGCAAGG
NK9	TCAATTAGGAGTAATTTTATGGTGAGCAAGG
NK48	TTGAGCTCTTACTTGTACAGCTCGTCCATGCC
NK78	ATGCCGTCTGAAGATGAACCAACTGCCACCTAAGG
NK83	TTGAGTCTTCCGACCCAATCAACACACCCGATAC
NK134	TTTCAATTAGGAGTAATTTTATGCGTAAAGGAGAAGAACTTTTCAC
NK135	AGTTCTTCTCCTTTACGCATAAAATTACTCCTAATTGAAAGGG
NK179	CAGTCGATTGGCTGAGCTCATGAGTATGTTAACGCGTAAATTCAAAAATC TCAAATTCC
NK180	GATAGAGTCAAGCCTCACGGGCAATTTTTTATTTTTTAAAAAGCTCCGTT TTCTTGG
NK181	CCAAGAAAACGGAGCTTTTTAAAAAATAAAAAATTGCCCGTGAGGCTTG ACTCTATC
NK182	GGAATTTGAGATTTTTGAATTTACGCGTTAACATACTCATGAGCTCAGCC AATCGACTG
NK199	ATGCCGTCTGAAACGACTGGATAAACCGCACATTCAAAGC
NK202	ATGCCGTCTGAATTGCTATGTTGGTTTAGGCTGACACG
NK203	GGCATGGATGAACTATACAAATAGGTGCTATAATTATACTAATTTTATAA GGAGG
NK204	CCTCCTTATAAAATTAGTATAATTATAGCACCTATTTGTATAGTTCATCCA TGCC
JB109	ATGCCGTCTGAAAGTCTTGGTAGGCGGGAAGGG
PILRBS	GGCATTTCCCCTTTCAATTAGGAG
SP3A	CCGGAACGGACGACCCCG

Table S2 List of primers

```
          ---------+---------+---------+---------+---SVR---+---------+---------+---------+
                   10        20        30        40        50        60        70        80
          ---------+---------+---------+---------+---------+---------+---------+---------+
Reference GCCGTCACCGAGTATTACCTGAATCACGGCAAATGGCCGGAAAACAACACTTCTGCCGGCGTGGCATCCCCCCCCA'CCG          80
pilS7c1   GCCGTCACCGAGTATTACCTGAATCACGGCATATGGCCGAAAGACAACACTTCTGCCGGCGTGGCATCCCCCCCCT'CCG          80
pilS6c1   GCCGTCACCGAATATTACCCGAATAACGGCAAATGGCCCGCCGACAACGGCGCTGCCGGCGTGGCATCTTCTTCAT''''          80
pilS6c3   '''''''''''''''''''''AATCACGGCAAATGGCCGGAAAACAAG'''CCTGCCGGCGTGGCATCCCCCGCCT'CCG          80
pilS5c1   GCCGTCACCGAGTATTACCTGAATCACGGCGAATGGCCCAAAGACAACGACTCTGCCGGCGTGGCATCCGCTTCAA''''          80
pilS1c5   GCCGTTACCGAGTATTACCTGAATCACGGCGAATGGCCCAAAGACAACGGCTCTGCCGGCGTGGCATCCGCTTCAA''''          80
pilS1c4   '''GTTGCCGGGTATTGCCTGAATCACGGCGAATGGCCGGAAGACAACACTTCTGCCGGCGTGGCATCCCCCCCAC''CG          80
pilS1c1   GCCGTTGCCGGGTATTGCCCGAATCACGGCAAATGGCCGGAAAACAACACTTCTGCCGGCGTGGCATCCCCCCCCCTCCG          80


          ---------+---------+---------+---------+---SVR---+---------+---------+---------+
                   90        100       110       120       130       140       150       160
          ---------+---------+---------+---------+---------+---------+---------+---------+
Reference ACATCAAAGGCAAATATGTTAAAGAGGTTGAAGTTAAAAACGGCGTCGTTACCGCCACAATGCTTTCAAGCGGCGTAAAC         160
pilS7c1   ACATCAAAGGCAAATATGTTCAAAGCGTTACGGTCGCAAACGGCGTCGTTACCGCCCAAATGAAATCAGACGGCGTAAAC         160
pilS6c1   CAATCAAAGGCAAATATGTTAAGGAAGTTAAAGTCGAAAACGGCGTCGTCACCGCCACAATGAATTCAAGCAACGTAAAC         160
pilS6c3   ACATCAAAGGCAAATATGTTCAAAGCGTTACGGTCGCAAACGGCGTCGTTACCGCCCAAATGAAATCAGACGGCGTAAAC         160
pilS5c1   AAATCATAGGCAAATATGTTAAGCAAGTTGAAGTCAAAAACGGCGTCGTTACCGCCCAAATGAAATCAGACGGCGTAAAC         160
pilS1c5   AAATCATAGGCAAATATGTTAAGGAAGTTAAAGTCGAAAACGGCGTCGTCACCGCCCAAATGGCTTCAAGCAACGTAAAC         160
pilS1c4   ACATCAAAGGCAAATATGTTAAAAGCGTTACGGTCGCAAAAGGCGTCGTCACCGCCGAAATGGCTTCAACCGGCGTAAAC         160
pilS1c1   ACATCAAAGGCAAATATGTTAAAGAGGTTGAAGTTAAAAACGGCGTCGTTACCGCCACAATGCTTTCAAGCGGCGTAAAC         160


          ---------+---------+---SVR---+---------+--|-------+---cys1--+---------+---------+
                   170       180       190       200|      210       220       230       240
          ---------+---------+---------+---------+--|-------+---------+---------+---------+
Reference AATGAAATCAAAGGCAAAAAACTCTCCCTGTGGGCCAAGCGT|GAAAACGGTTCGGTAAAATGGTTCTGCGGACAGCCGGT          240
pilS7c1   AAAGAAATCAAAAACAAAAAACTCTCCCTGTGGGCCAAGCGT|GAAAACGGTTCGGTAAAATGGTTCTGCGGACAGCCGGT          240
pilS6c1   AAAGAAATCAAAGACAAAAGACTCTCCCTGTGGGGCAGGCGT|GAAAACGGTTCGGTAAAATGGTTCTGCGGACAGCCGGT          240
pilS6c3   AAAGAAATCAAAAACAAAAAACTCTCCCTGTGGGCCAGGCGT|GAAGCCGGTTCGGTAAAATGGTTCTGCGGACAGCCGGT          240
pilS5c1   AAAGAAATCAAAAACAAAAAACTCTCCCTGTGGGCCAAGCGT|GAAAACGGTTCGGTAAAATGGTTCTGCGGACAGCCGGT          240
pilS1c5   AAAGAAATCAAAGACAAAAAACTCTCCCTGTGGGCCAAGCGT|GAAAACGGTTCGGTAAAATGGTTCTGCGGACAGCCGGT          240
pilS1c4   AATGAAATCAAAGGCAAAAAACTCTCCCTGTGGGCCAAGCGT|CAAGACGGTTCGGTAAAATGGTTCTGCGGACAGCCGGT          240
pilS1c1   AATGAAATCAAAGGCAAAAAACTCTCCCTGTGGGCCAGGCGT|GAAAACGGTTCGGTAAAATGGTTCTGCGGACAGCCGGT          240


          -|--------+---------+---------+---HVL---+---------+---------+---------+------|---+
           |        250       260       270       280       290       300       310  |  320
          -|--------+---------+---------+---------+---------+---------+---------+------|---+
Reference T|ACGCGCGCC'''''''''''''''GCCAAAGACGACGACGCCGTCACCGCCGAC'''GGCAACAACAAAATCGAC|ACCA          320
pilS7c1   T|ACGCGCGCC'''''''''''''''GCCAAAGACGACGACGCCGTCACCGCCGAC'''GGCAACAACAAAATCGAC|ACCA          320
pilS6c1   T|ACGCGCA'''''''''ACGCCAACGACGAC'''ACCGTCACCGCCGACGGCACCGGCAACGACGGCAAAATCGAC|ACCA          320
pilS6c3   T|ACGCGCGAC''''''''''''AAAGCCGTCACCGACGACGCCGTCAAAGACGTCACCGGCAACGACAAAATCGAA|ACCA          320
pilS5c1   T|ACGCGCA'''''''''ACGCCAAAGCCAAC'''GACACCGTTGCCGCCGACGGCACCGGCAACGACAAAATCGAA|ACCA          320
pilS1c5   T|AAGCGCACCGAAGCCAACGCCAAAGCCGGCACCGACGACGTCGCCAAAGACGACACCGCCGGCACCAAAATCGAC|ACCA          320
pilS1c4   T|AAGCGCGACG'''CCGGCGCCAAAACCGGCGCCGACGACGTCAAAGCCGACGGCAAAGACACCGACAAAATCAAC|ACCA          320
pilS1c1   T|ACGCGCGCC'''''''''''''''AAAGCCGACGCCGACGCCGACGCCGCCGGCAAAGACACCACCAACATCGAC|ACCA          320
```

```
          ---cys2--+---------+------|---+---HVT---+---------+---
                   330       340    |   350       360       370
          ---------+---------+------|---+---------+---------+---
Reference AGCACCTGCCGTCAACCTGCCGCGAT|AAGGCATCTGATGCCAAATGA''''''                                  373
pilS7c1   AGCACCTGCCGTCAACCTGCCGCGAC|ACTTCATCTGCCGGTAAG'''''''''                                  373
pilS6c1   AGCACCTGCCGTCAACCTGCCGCGAC|ACTTCATCA''GCCGGTAAG'''''''                                  373
pilS6c3   AGCACCTGCCGTCAACCTGCCGT'''|'''''''''''''''''''''''''''                                  373
pilS5c1   AGCACCTGCCGTCAACCTGCCGCGAT|AACT''TTGATGCCAGCTGA'''''''                                  373
pilS1c5   AGCACCTGCCGTCAACCTGCCGCGAT|GAATCATCGTTGCCGGGTATTGCCTGA                                  373
pilS1c4   AGCACCTGCCGTCAACCTGCCGCGAT|AAATCATCTGCCGTT''''''''''''                                  373
pilS1c1   AGCACCTGCCGTCAACCTGCCGCGAT|GAATCATCTGCCGTTTGCACGAAAC''                                  373
```

Table S3 Sequences of *pilS* aligned to reference *pilE* sequence using MEGA7 software [6]. Only the semivariable region SVR, the conserved cys1 and cys2 regions, the hypervariable loop HVL and the hypervariable tail HVT are shown.

References

1. Kouzel, N., Oldewurtel, E.R. & Maier, B. Gene Transfer Efficiency in Gonococcal Biofilms: Role of Biofilm Age, Architecture, and Pilin Antigenic Variation. *Journal of bacteriology* **197**, 2422-31 (2015).
2. Wolfgang, M., van Putten, J.P., Hayes, S.F., Dorward, D. & Koomey, M. Components and dynamics of fiber formation define a ubiquitous biogenesis pathway for bacterial pili. *The EMBO journal* **19**, 6408-18 (2000).
3. Oldewurtel, E.R., Kouzel, N., Dewenter, L., Henseler, K. & Maier, B. Differential interaction forces govern bacterial sorting in early biofilms. *eLife* **4**(2015).
4. Koomey, J.M. & Falkow, S. Cloning of the recA gene of Neisseria gonorrhoeae and construction of gonococcal recA mutants. *J Bacteriol* **169**, 790-5 (1987).
5. Tonjum, T., Freitag, N.E., Namork, E. & Koomey, M. Identification and characterization of pilG, a highly conserved pilus-assembly gene in pathogenic Neisseria. *Molecular microbiology* **16**, 451-64 (1995).
6. Kumar, S., Stecher, G. & Tamura, K. MEGA7: Molecular Evolutionary Genetics Analysis Version 7.0 for Bigger Datasets. *Molecular Biology and Evolution* **33**, 1870-1874 (2016).
7. Vink, C., Rudenko, G. & Seifert, H.S. Microbial antigenic variation mediated by homologous DNA recombination. *FEMS Microbiol Rev* **36**, 917-48 (2012).

Supplementary Figures

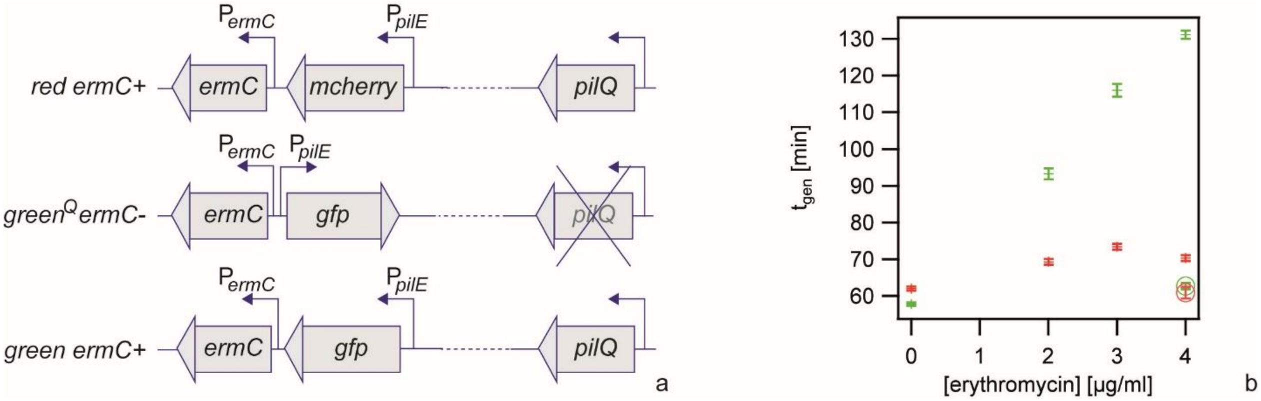

Figure S1 Tuning exponential growth rates and bacterial interaction force independently. a) Different constructs used. b) Generation times of the strains shown in a) as a function of the concentration of erythromycin. Red cross: *red ermC+*, green cross: *green*^{Q} *ermC-*, red circle: *red ermC+* (control), green circle: *green ermC+* (control). The generation times varied slightly between days (red cross and red circle) and thus different strains were always grown on the same plate for comparison. Error bars: standard errors of the mean, N = (166 – 1206) colonies.

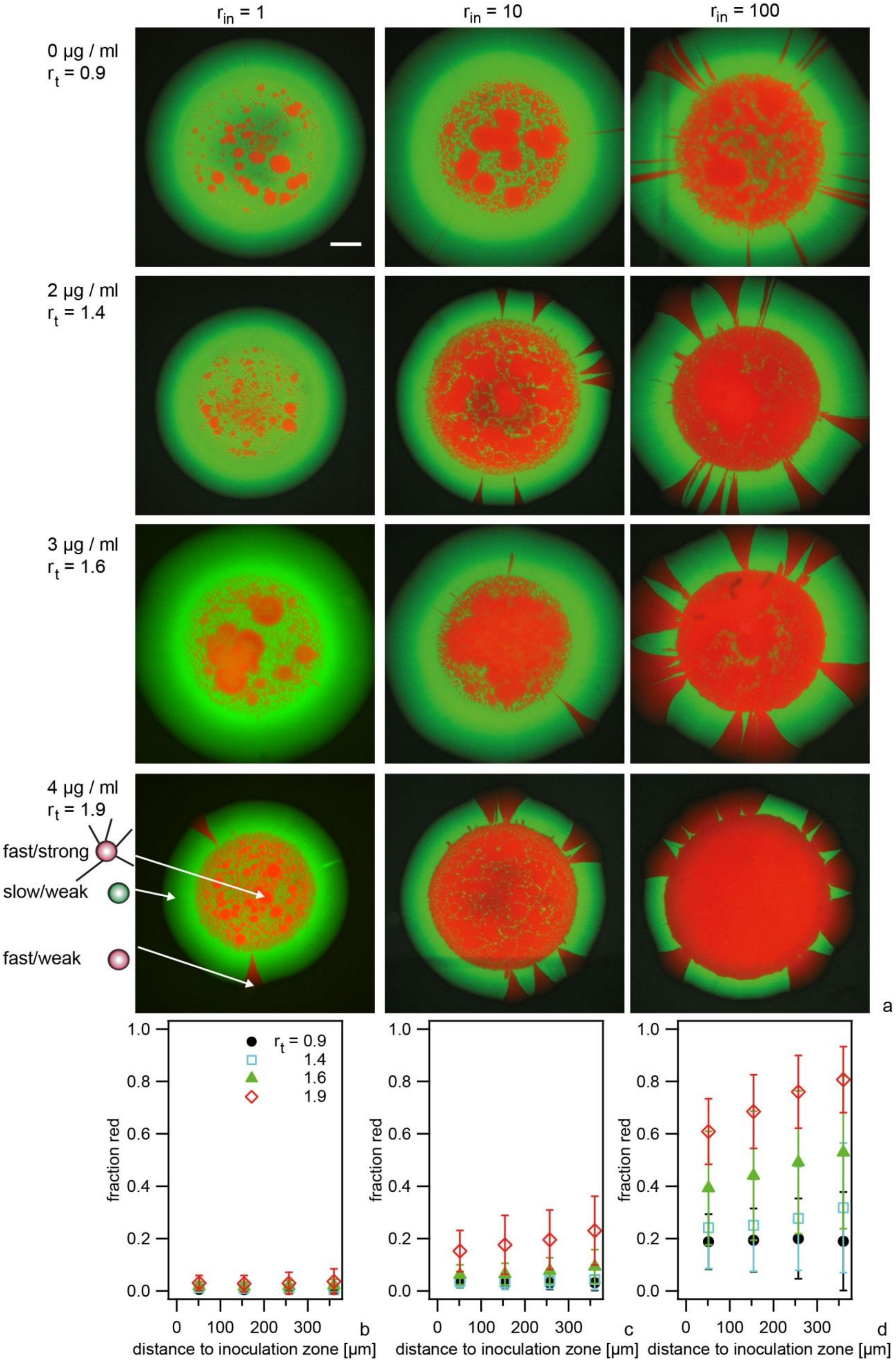

r_{in} = 1
r_{in} = 10
r_{in} = 100
0 µg / ml
r_t = 0.9
2 µg / ml
r_t = 1.4
3 µg / ml
r_t = 1.6
4 µg / ml
r_t = 1.9
fast/strong
slow/weak
fast/weak
a
fraction red
r_t = 0.9
1.4
1.6
1.9
distance to inoculation zone [µm]
b
c
d

Figure S2 Competition between initially strongly interacting and weakly interacting cells at varying exponential growth rates. a) Examples of mixed macrocolonies after 68 - 70 h at varying concentrations of antibiotics. The ratio r_{in} on top indicates the ratio between *red ermC+* $recA_{ind}$ (Ng065) and $green^{Q}$ *ermC-* $recA_{ind}$ (Ng118) inoculated onto the agar plate. $r_t = t_{gen}(green^{Q}\ ermC-)/t_{gen}(red\ ermC+)$. Scale bar: 500 µm. Fraction of *red ermC+* cells within expanding front as a function of distance from the inoculum for [*red ermC+*] *:* [$green^{Q}$ *ermC-*] of b) r_{in}= 1, c) r_{in}= 10, d) r_{in}= 100. The error bars are the standard deviations obtained from 16 macrocolonies for each condition.

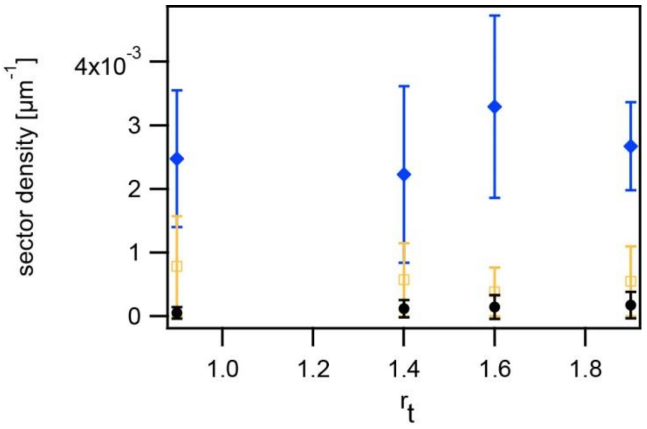

Figure S3 Sector density for competition between initially strongly interacting *red ermC+ recA*$_{ind}$ (Ng065) and weakly interacting *green*^{Q} *ermC- recA*$_{ind}$ (Ng118) cells at varying exponential growth rates. The number of sectors formed by *red ermC+* that reached the expanding front after (68 - 70) h was normalized to the circumference of the front. Ratio of generation times $r_t = t_{gen}$*(green*^{Q} *ermC-)/*t_{gen}*(red ermC+).* Inoculation ratios between *red ermC+* and *green*^{Q} *ermC-* were black circles: r_{in}= 1, orange squares: r_{in}= 10, blue diamonds: r_{in}= 100. The error bars are the standard deviations obtained from 16 macrocolonies for each condition.

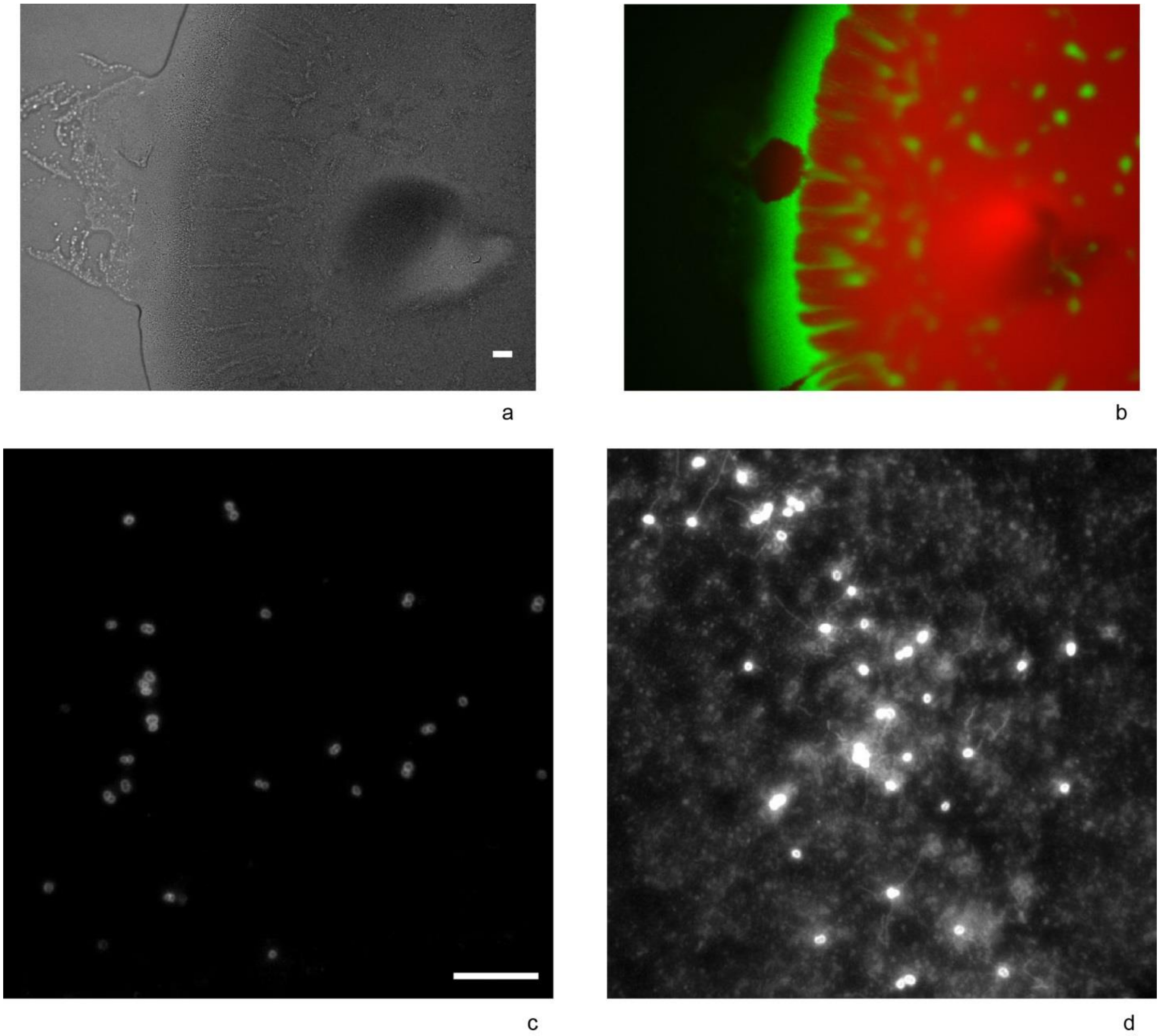

Figure S4 Status of piliation in inoculation zone and in the area of outgrowth in the presence of erythromycin. Piliated *red ermC+* (Ng065) and *green*^{Q} *ermC-* (Ng118) were inoculated at a ratio of 1 to 10 on 4 µg/ml erythromycin. Bacteria were picked from the area of outgrowth (a, b) or from the inoculation zone, grown overnight and selected for red fluorescence. Subsequently, bacteria were immobilized on glass cover slide and stained using immunofluorescence with a primary antibody against PilE. c) Area of outgrowth, d) inoculation zone. Scale bars: 50 µm.

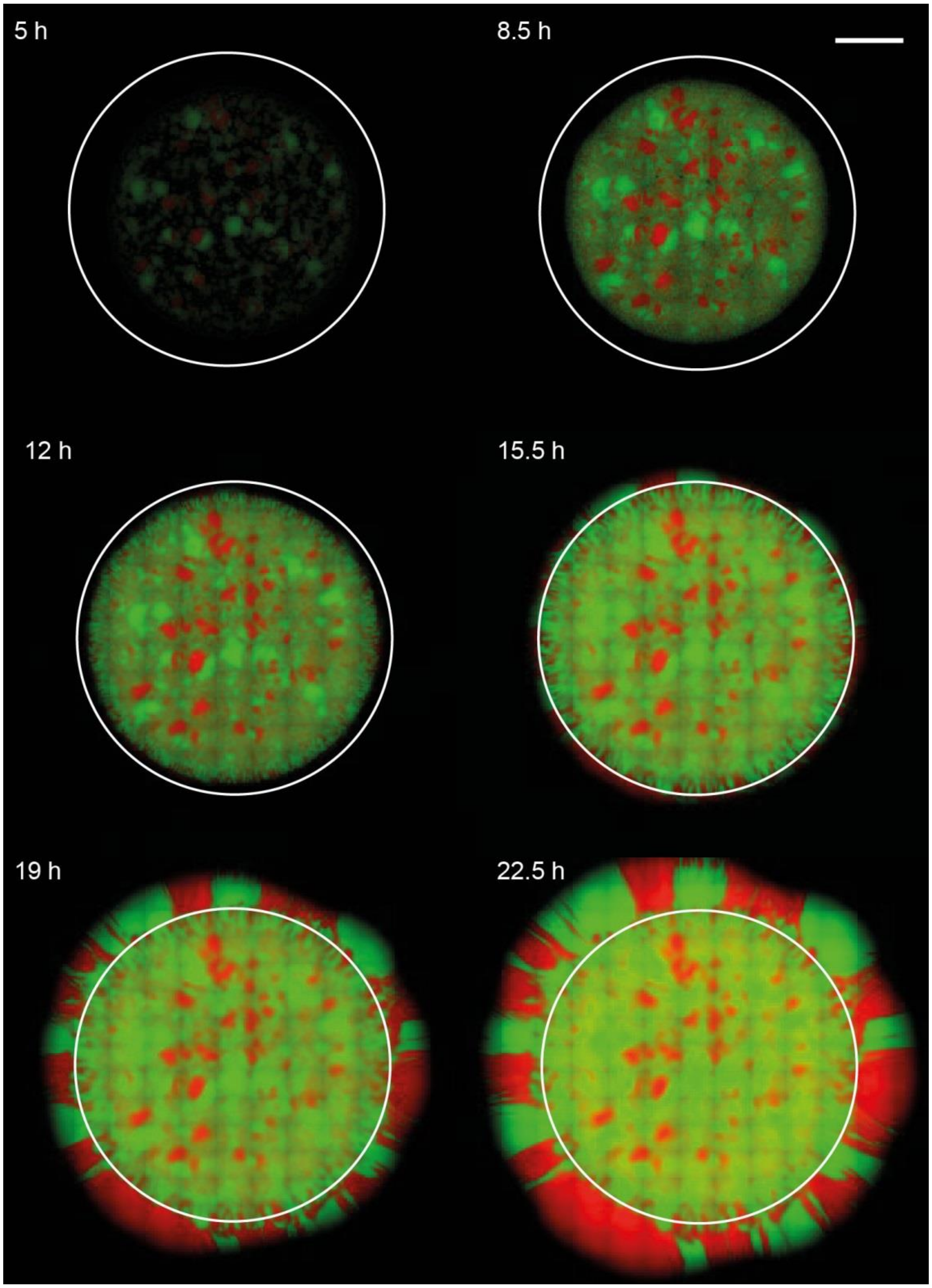

Figure S5 Time lapse of colony growth. A droplet of piliated *red wt* (Ng106) and *green wt* (Ng165) was inoculated. A region of interest of this time lapse is shown in Fig. 5. Please note that the front closes at ~ 5 h, but this process is not visible at this low resolution. Scale bar: 500 µm. To guide the eye, the white circle (constant radius) was overlaid.

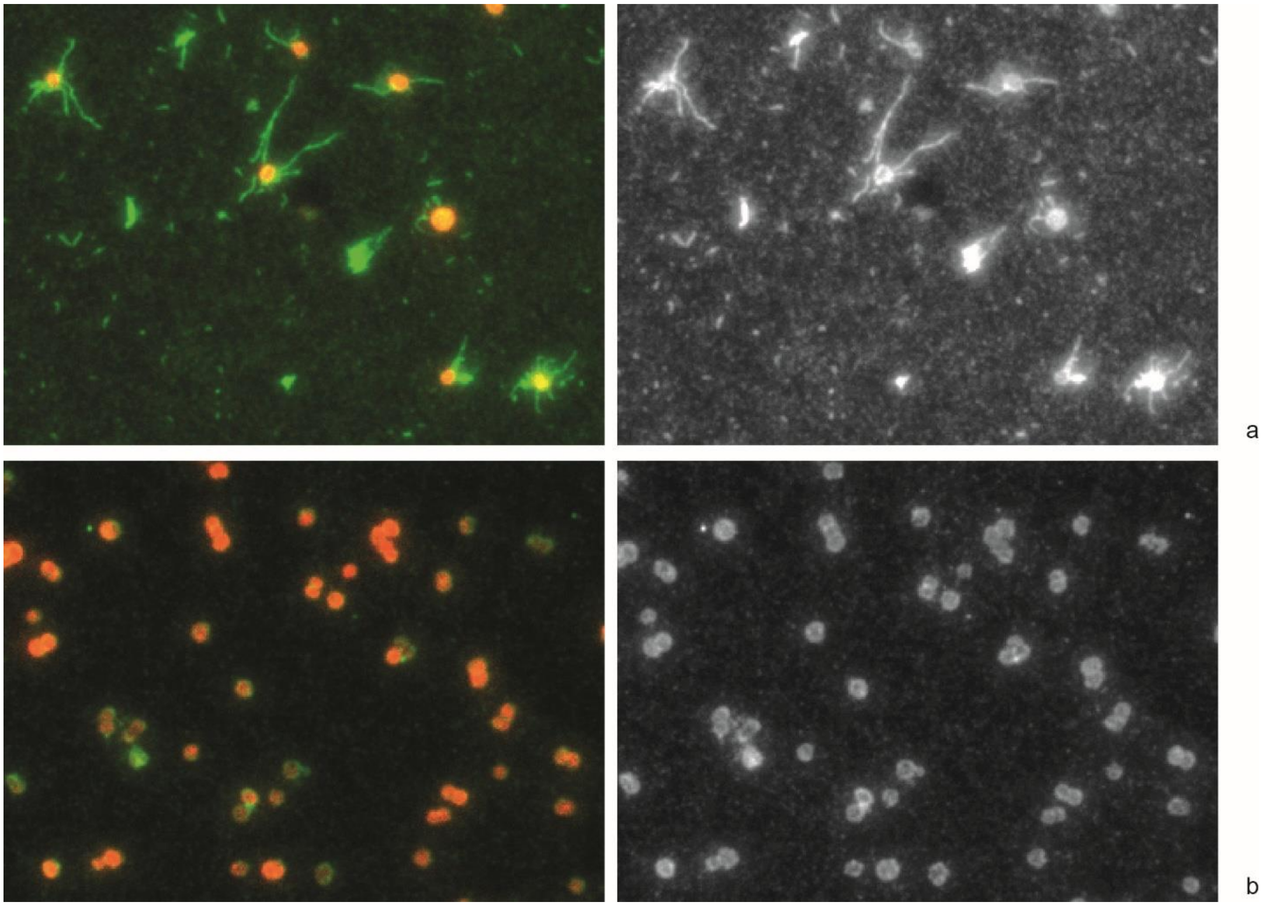

Figure S6 Status of piliation in inoculation zone and in the area of outgrowth. Aggregation of piliated gonococci leads to three-dimensional spherical colonies whereas non-interacting non-piliated gonococci form more flat colonies of larger diameter [7]. To confirm our ability to clearly distinguish between colonies on agar plates of piliated and unpiliated gonococci by visual inspection of colonies on agar plates, we selected individual a) piliated and b) non-piliated colonies (Ng165), immobilized them on a cover slide and stained using immunofluorescence with a primary antibody against PilE. Red: signal from cytoplasmic YFP, green, immunofluorescence detecting PilE. The imaging parameters and the contrast are equal for a) and b). One typical example from three independent experiments is shown.

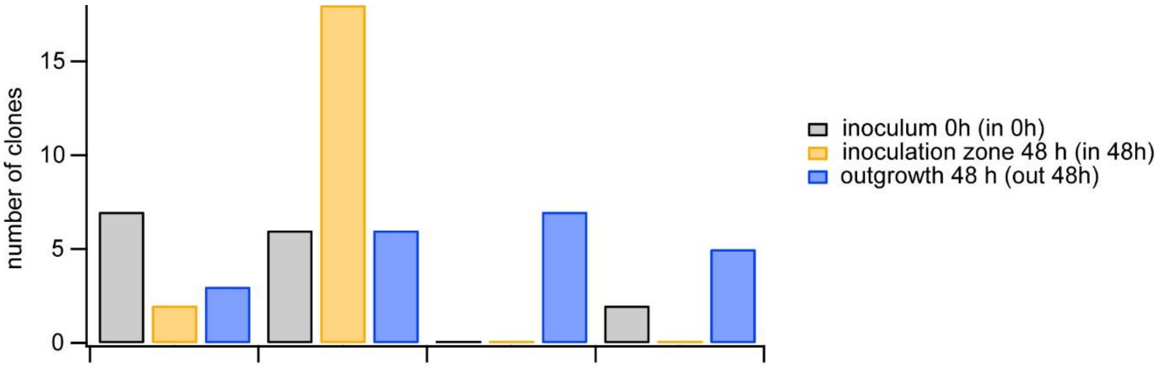

Fisher's p-values between	full length	full length, av	truncated, pv	no PCR
in 0h - in 48 h	0.02	0.003		
in 0h - out 48 h			0.03	
in 48h - out 48 h		10^{-4}	0.009	0.05

Figure S7 Significance levels of sequencing data presented in Fig. 7. *red wt* (Ng106) and *green wt* (Ng165) were picked from the initial inoculum, the inoculation zone (48 h), and the outgrowth (48 h), respectively. After dilution and growth on agar plates, individual colonies (clones) were picked and *pilE* was sequenced. Sequences were categorised into full length: most abundant sequence in inoculum; full length, av: sequence change mappable to *pilS* sequence; truncated, pv: length change in poly-C sequence causing premature stop-codon; no PCR: PCR amplification did not result in a product. Upper figure: Total number of clones per category of inoculum, inoculation zone and outgrowth, respectively. Lower table: P-values determined from Fisher's exact test. Only significance levels $p < 0.05$ are shown.

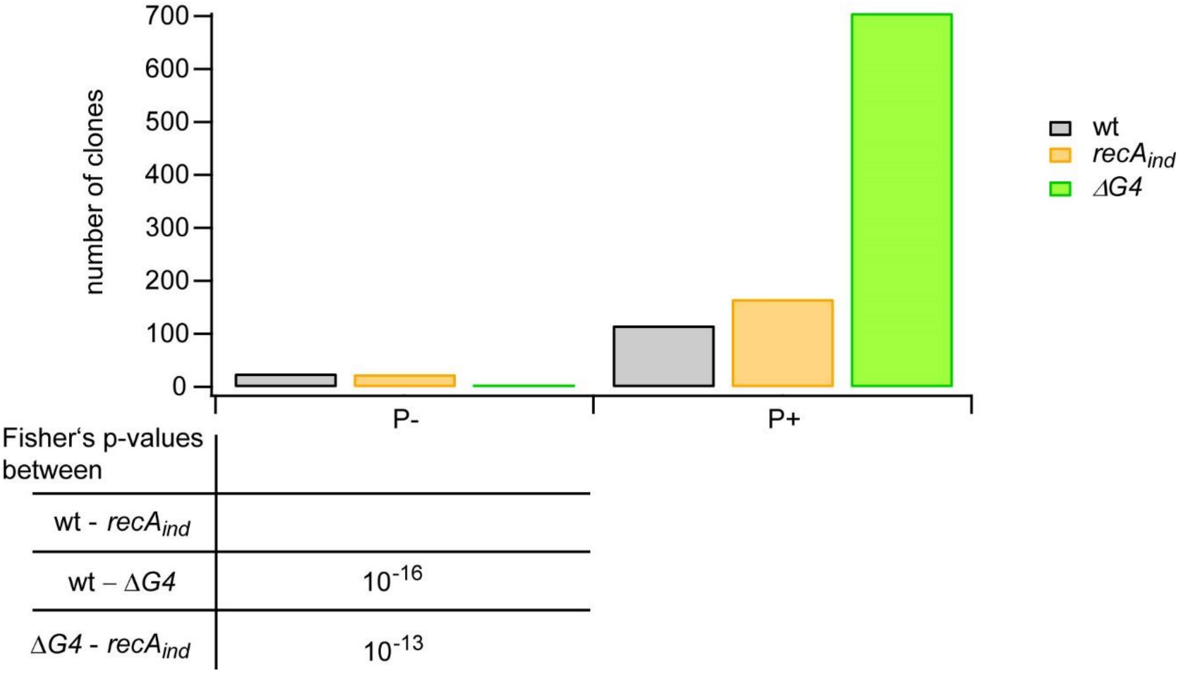

Fisher‘s p-values between	P-
wt - *recA*$_{ind}$	
wt – Δ*G4*	10^{-16}
Δ*G4* - *recA*$_{ind}$	10^{-13}

Figure S8 Significance levels of sequencing data presented in Fig. 8c. A droplet of differentially labeled strains was inoculated onto an agar plate and colonies were grown for 48 h. Upper figure: Total number of non-piliated P- and piliated P+ clones in the area of inoculation of the 48 h colony were determined by colony morphology. Lower table: P-values determined from Fisher's exact test. Only significance levels $p < 0.05$ are shown.

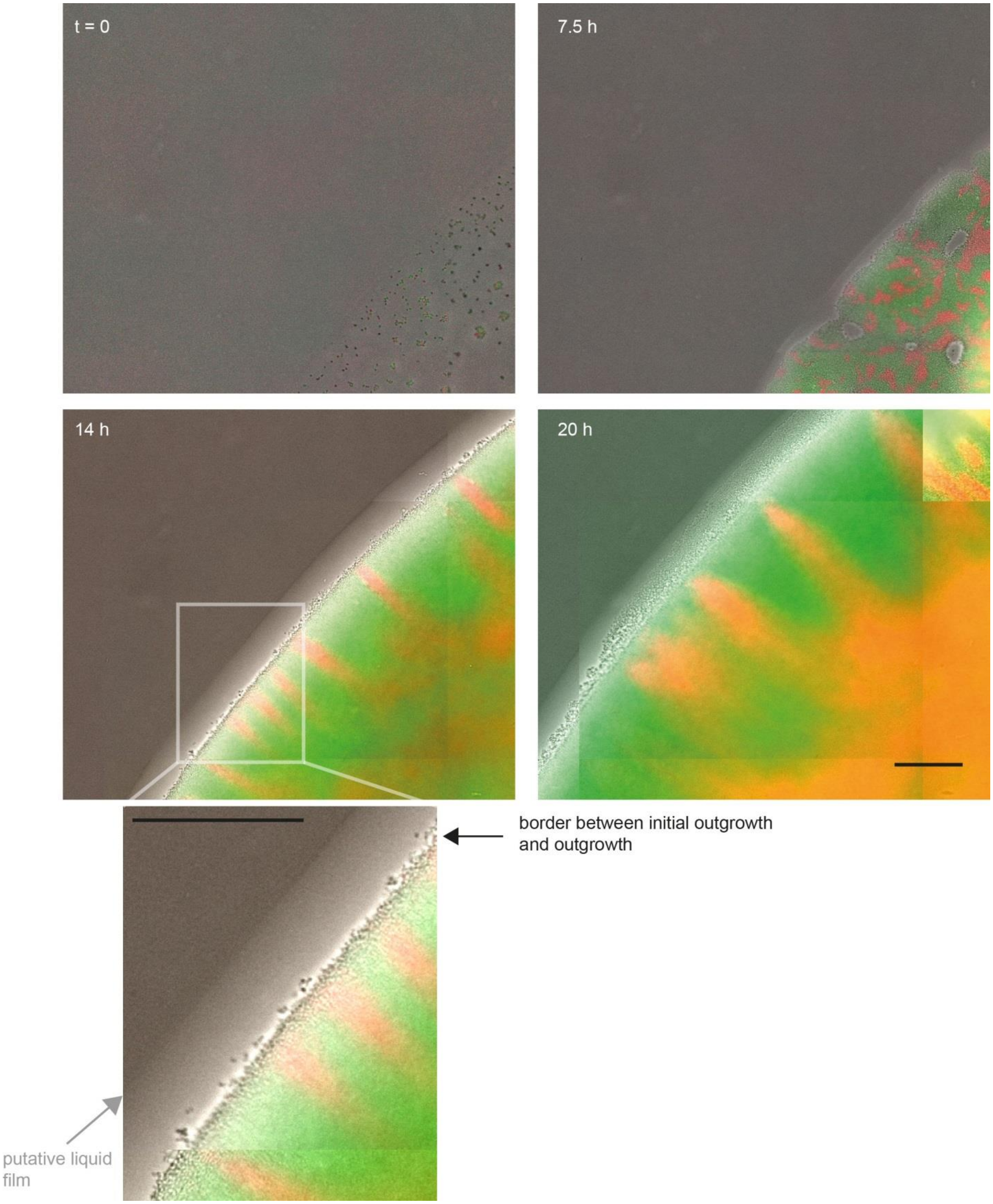

Figure S9 Dynamics of expanding front. Time lapse of growing macrocolony (region of interest of Movie S1). A droplet of piliated *green* Δ*G4* (Ng169) and *red* Δ*G4* (Ng170) was inoculated. Overlay between brightfield image and fluorescence images. Scale bar: 50 µm.

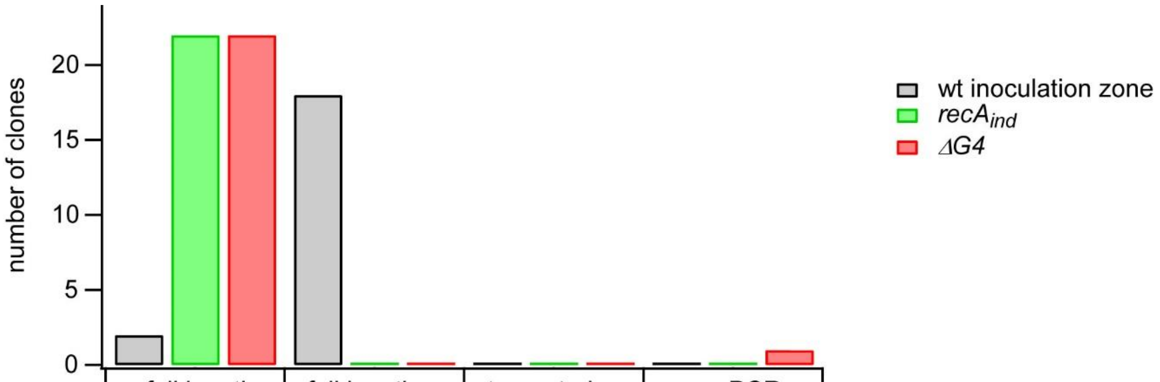

Fisher's p-values between	full length	full length, av	truncated, pv	no PCR
wt - *recA*$_{ind}$	10^{-9}	10^{-9}		
wt – Δ*G4*	10^{-8}	10^{-9}		
Δ*G4* - *recA*$_{ind}$				

a

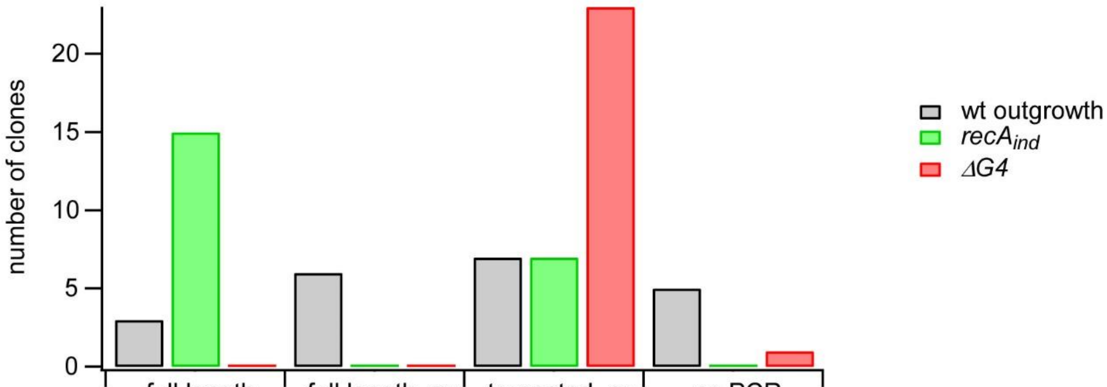

Fisher's p-values between	full length	full length, av	truncated, pv	no PCR
wt - *recA*$_{ind}$	10^{-3}	0.009		0.02
wt – Δ*G4*		0.007	10^{-5}	
Δ*G4* - *recA*$_{ind}$	10^{-6}		10^{-5}	

b

Figure S10 Significance levels of sequencing data presented in Fig. 9. Pilin variation in antigenic variation deficient strains**.** Cells were picked from a) inoculation zone, and b) outgrowth, respectively. After dilution and growth on agar plates, individual colonies (clones) were picked and *pilE* was sequenced. Sequences were categorised as full length: most abundant sequence in inoculum; full length, av: sequence change mappable to *pilS* sequence; truncated, pv: length change in poly-C sequence causing premature stop-codon; no PCR: PCR amplification did not

result in a product. Upper figure: Total number of clones per category of inoculum, inoculation zone and outgrowth, respectively. Lower table: P-values determined from Fisher's exact test. Only significance levels $p < 0.05$ are shown.

Supplementary Movie Captions

Movie S1 Live range expansion. A droplet of piliated *red wt* (Ng106) and *green wt* (Ng165) was inoculated.

Movie S2 Live range expansion (high resolution detail of Movie S1). A droplet of piliated *red wt* (Ng106) and *green wt* (Ng165) was inoculated. Scale bar: 50 µm, time in [hours:minutes].
